# Two Sugarcane *Expansin* Protein-Coding Genes Contribute to Stomatal Aperture Associated with Structural Resistance to Sugarcane Smut

**DOI:** 10.3390/jof10090631

**Published:** 2024-09-03

**Authors:** Zongling Liu, Zhuoxin Yu, Xiufang Li, Qin Cheng, Ru Li

**Affiliations:** 1 State Key Laboratory for Conservation and Utilization of Subtropical Agro-Bioresources, College of Life Science and Technology, Guangxi University, Nanning 530004, China; 2 College of Basic Medical Sciences, Youjiang Medical University for Nationalities, Baise 533000, China; 3 Guangxi Subtropical Crops Research Institute, Nanning 530001, China

**Keywords:** Expansin protein, RNA-seq, resistance, stomata, sugarcane smut

## Abstract

*Sporisorium scitamineum* is a biotrophic fungus responsible for inducing sugarcane smut disease that results in significant reductions in sugarcane yield. Resistance mechanisms against sugarcane smut can be categorized into structural, biochemical, and physiological resistance. However, structural resistance has been relatively understudied. This study found that sugarcane variety ZZ9 displayed structural resistance compared to variety GT42 when subjected to different inoculation methods for assessing resistance to smut disease. Furthermore, the stomatal aperture and density of smut-susceptible varieties (ROC22 and GT42) were significantly higher than those of smut-resistant varieties (ZZ1, ZZ6, and ZZ9). Notably, *S. scitamineum* was found to be capable of entering sugarcane through the stomata on buds. According to the RNA sequencing of the buds of GT42 and ZZ9, seven *Expansin* protein-encoding genes were identified, of which six were significantly upregulated in GT42. The two genes c111037.graph_c0 and c113583.graph_c0, belonging to the α-*Expansin* and β-*Expansin* families, respectively, were functionally characterized, revealing their role in increasing the stomatal aperture. Therefore, these two sugarcane *Expansin* protein-coding genes contribute to the stomatal aperture, implying their potential roles in structural resistance to sugarcane smut. Our findings deepen the understanding of the role of the stomata in structural resistance to sugarcane smut and highlight their potential in sugarcane breeding for disease resistance.

## 1. Introduction

Sugarcane is a widely cultivated crop that serves as a valuable source of sugar, fiber, wax, biofuel, and other products [1]. *Sporisorium scitamineum* is a basidiomycete fungus causing sugarcane smut disease, seriously affecting the yield and quality of sugarcane. Initially, teliospores of *S. scitamineum* fall on the surface of sugarcane buds and germinate under appropriate conditions [2]. Meiosis then occurs once the germ tube enters the bud tissue, leading to the formation of haploid sporidia. Finally, haploid cells of different mating types fuse and form dikaryotic mycelium, which can invade sugarcane tissues [3]. *S. scitamineum* expands within the sugarcane as growth progresses, eventually causing black whip-like structures at the top of the stem [3].

The mechanisms of smut resistance can be classified into three categories: structural, biochemical, and physiological resistance [4,5]. Structural resistance refers to a sugarcane-inherent structure that play roles in *S. scitamineum* defense [6]. Biochemical and physiological resistance refer to sugarcane responses that defend against *S. scitamineum* invasion [2]. The evaluation of sugarcane smut resistance differs between field incidence rates and artificial inoculation, as the latter destructs the outer structure of buds, allowing for direct invasion by *S. scitamineum* into the bud interior [4]. This suggests that structural components may play a crucial role in conferring smut resistance. The germination process of teliospores can be influenced by glycoside flavonoid compounds present on the bud surface. Typically, upon germination, the germ tube of *S. scitamineum* penetrates sugarcane tissue by forming an appressorium [2,7,8]. If there are openings in the sugarcane bud, the germ tube of *S. scitamineum* can directly and easily enter the sugarcane tissue, resulting in a higher susceptibility to smut infection. For example, sugarcane inoculated with *S. scitamineum* through acupuncture inoculation causes openings, resulting in a higher incidence rate of smut and a greater presence of *S. scitamineum* mycelium within tissues [9,10].

Stomata are natural openings on the surface of plants that primarily facilitate gas exchange with the external environment [11]. Stomata also serve as an important channel for microorganisms to enter plants, including bacteria, fungi, actinomycetes, and yeast [12,13]. Some plant pathogens have been reported to enter plants through the stomata. For example, *Fusarium graminearum* can invade plants by secreting cell wall-degrading enzymes and utilizing stomata as an entry route [14]. The germ tube of *Puccinia striiformis* grows toward and enters wheat stomata, subsequently forming vesicles along with infected hyphae and haustoria inside the stoma [15]. *P. striiformis* employs a similar mechanism to invade *Phaseolus vulgaris* [16,17]. In addition, *Plasmopara viticola* recognizes the stomatal position on grape leaves to form an appressorium for further invasion [18]. Therefore, plant stomatal characteristics can serve as an indicator of plant disease resistance. Stomatal density and stomatal aperture have been observed to be significantly higher in susceptible wheat cultivars infected by *Erysiphe graminis* compared to resistant cultivars; however, no significant differences are observed in terms of stomatal area or perimeter [19]. Stomata serve as open structures in sugarcane that may facilitate invasion by *S. scitamineum* germ tubes and thus represent a form of structural resistance. In plants, bHLH transcription factors, including SPCH, MUTE, and FAMA, mainly regulate stomatal development [20,21,22], and *Expansin* proteins mainly regulate stomatal aperture [23]. Therefore, these genes may be involved in plant disease resistance by regulating stomata. In this study, the relationship between the stomatal characteristics of sugarcane buds and sugarcane smut resistance was investigated. Significant differences were observed in the stomatal features of smut-resistant and smut-susceptible sugarcane varieties, and the invasion of *S. scitamineum* through the bud stomata was quantified. Through RNA-seq analysis, two genes encoding *Expansin* proteins that affect stomatal aperture were identified and functionally characterized. This study provides a deeper understanding of the structural resistance mechanisms involved in sugarcane’s defense against smut and highlights their potential in sugarcane breeding for disease resistance.

## 2. Materials and Methods

### 2.1. Sugarcane Varieties and S. scitamineum Inoculation

Three smut-resistant varieties, ZZ1, ZZ6, and ZZ9, and two smut-susceptible varieties, ROC22 and GT42, were cultivated with routine management at the Guangxi University Sugarcane Germplasm Nursery (22.5° N, 107.7° E), Guangxi province, China. The single bud segments of ZZ9 and GT42, which exhibited the highest smut-resistant and smut-susceptible characteristics in the field, were collected and soaked in running water for 1 d for further *S. scitamineum* inoculation. *S. scitamineum* teliospores were collected from the smut whip of ROC22 in the field of the Guangxi University Sugarcane Germplasm Nursery in September 2021. The teliospores were pre-treated, then stored at 4 °C before use [24]. Two inoculation methods were conducted, namely, puncture and soaking inoculation [10]. For puncture inoculation, aliquots of 2 μL suspension of *S. scitamineum* teliospores (5 × 10^6^ spores/mL) were injected into the buds of segments. For soaking inoculation, the segments were soaked in an *S. scitamineum* teliospore suspension (5 × 10^6^ spores/mL) for 30 min. Sterile water was used as the control. Three inoculated sugarcane segments were planted in a barrel with matrix soil and cultivated in a greenhouse with routine management at 28 °C, with a 12 h/12 h light and night cycle, and at 90% RH. The smut incidence was recorded 100 d after planting.

### 2.2. Scanning Electron Microscopy Observations

The stomatal characteristics of the buds were observed using scanning electron microscopy (SEM, FEI Quattro S, Thermo Fisher Scientific, Waltham, MA, USA) [6]. The outermost scales of the buds from eight-month-old sugarcane with identical growth conditions were collected and soaked in a 2.5% glutaraldehyde solution in 0.1 M phosphate buffer (pH 7.3) at 4 °C for over 24 h. The scales were then washed with 0.1 M phosphate buffer (pH 7.3) three times. Following this, the scales were gradient-dehydrated using an increasing gradient of 10%, 30%, 50%, 70%, 90%, and 100% alcohol. Each concentration treatment lasted for 10 min. Tert-butanol was then used to replace the anhydrous alcohol before freeze-drying all samples using a freeze dryer (FreeZone, Labconco, Kansas City, MO, USA) for 1 h. Once freeze-dried, the samples were coated with gold using a magnetron sputtering instrument (Sputter Coater 108, Cressington, Liverpool, UK) and then observed using SEM. Five buds of each variety were selected for the observations.

SEM was also employed to observe the invasion of sugarcane by *S. scitamineum* from the bud stomata. In particular, the middle healthy stem segments of five sugarcane varieties were cut into single bud segments. An aliquot of 10 μL suspension of *S. scitamineum* teliospores (5 × 10^6^ cfu/mL, 0.01% tween20) was dropped on the buds, which were placed at 28 °C and 80% RH for 24 h. The outermost scales of the buds, containing germinated *S. scitamineum* teliospores, were used for SEM observations according to the above method.

### 2.3. RNA Sequencing

RNA sequencing was performed to investigate the expression profiles of stomata-related genes. The outermost scales of 15 buds from eight-month-old GT42 and ZZ9 varieties with the same growth conditions were collected and frozen using liquid nitrogen. Sequencing of each sample was performed in three biological replications. All samples were sent to Beijing Biomarker Technologies Inc., Beijing, China, for RNA extraction using Trizol reagent and DNase I treatment; Illumina sequencing library construction was conducted using NEBNext^®^Ultra™ RNA Library Prep Kit for Illumina^®^ (NEB, Ipswich, MA, USA), and Illumina sequencing was performed using the Illumina Hiseq 2000 platform (Illumina, San Diego, CA, USA).

Raw data were filtered by fastp v18.0 [25] and the low-quality and connector-containing reads were removed. Trinity v2.5.1 [26] was used to assemble clean reads for obtaining unigenes. All unigenes were annotated using the eggNOG (http://eggnog-mapper.embl.de/, accessed on 7 June 2024) and Pfam databases (http://www.pfam.org/, accessed on 7 June 2024). The fragments per kilobase of exon model per million mapped fragments of each unigene was calculated using RSEM v1.2.15 [27]. Differentially expressed genes (DEGs) were analyzed using the DESeq R package v1.10.1 with FDR < 0.01 and FC ≥ 2. The stomata-related unigenes were identified based on the DEGs and annotations.

### 2.4. Reverse Transcription–Quantitative PCR

Four genes encoding *Expansin* proteins were selected for reverse transcription–quantitative PCR (RT-qPCR) according to FDR and log_2_FC values, namely, c121409.graph_c0, c103790.graph_c0, c111037.graph_c0, and c113583.graph_c0. The RT-qPCR primers were designed using the primer-BLAST tool (https://www.ncbi.nlm.nih.gov/tools/primer-blast/, accessed on 7 June 2024) (Table S1). The remaining RNA of the RNA-seq was used for RT-qPCR analysis. BeyoRT™ III cDNA synthesis premix (5×) (with gDNA EZeraser) (D7185M, Beyotime, Shanghai, China) and ChamQ Universal SYBR qPCR Master Mix (Q711, Vazyme, Nanjing, China) were used for reverse transcription and qPCR following the manufacturer’s instructions. The housekeeping gene encoding glyceraldehyde-3-phosphate dehydrogenase (*GAPDH*) was used as the internal standard. All data were analyzed using the 2^−∆∆Ct^ method [28].

### 2.5. Protein Sequence Analysis of c111037.graph_c0 and c113583.graph_c0

*Expansin* proteins were categorized as α-Expansin, β-Expansin, Expansin-like A, and Expansin-like B families [29]. A total of 13 *Expansin* proteins (four α-Expansin, three β-Expansin, two Expansin-like A, two Expansin-like B, c111037.graph_c0, and c113583.graph_c0) were selected to conduct phylogenetic tree analysis. Sequence alignment analysis and phylogenetic tree construction were performed with MEGA7 [30] using the ClustalW and maximum likelihood methods, respectively. The conserved domains of the *Expansin* proteins were predicted by the Batch CD-Search Tool (https://www.ncbi.nlm.nih.gov/Structure/bwrpsb/bwrpsb.cgi, accessed on 7 June 2024). The sequence alignments and phylogenetic trees were visualized using GENEDOC v2.7 [31] and TBtools v1.09 [32], respectively.

### 2.6. Vector Construction and Transient Expression in Nicotiana benthamiana

The primers for the ORFs (Open Reading Frames) of c111037.graph_c0 and c113583.graph_c0 were designed using SnapGene v4.2.4 for infusion cloning (Table S1). Amplicons were obtained using 2× Phanta Max Master Mix (Vazyme, China), with the cDNA transcribed from the RNA using BeyoRT™ III cDNA synthesis premix (5×) (with gDNA EZeraser) as the template. The vector pCAMBIA3300-CaMV 35S was linearized by *Bam* HI and *Sac* I (Thermo Fisher Scientific, USA). The recombinant vectors of 35S::c111037.graph_c0 and 35S::c113583.graph_c0 were constructed using 2× Seamless Cloning Mix (Beyotime) and transformed into *Escherichia coli* chemical competent T1 cells (Vazyme). The correct insertion of constructs was validated through DNA sequencing and subsequently extracted using a FastPure Plasmid Mini Kit (Vazyme).

The vectors of 35S::c111037.graph_c0 and 35S::c113583.graph_c0 were transformed into *Agrobacterium* chemical competent GV3101 cells (AngYuBio, Shanghai, China). The GV3101 cells harboring 35S::c111037.graph_c0 and 35S::c113583.graph_c0 were cultured in YEP medium (1% beef extract, 1% yeast extract, 0.5% NaCl, 50 μg/mL Kan, and 20 μg/mL Rif, at pH 7.0) at 28 °C for 36 h. The cells were then resuspended twice using an inoculation buffer (10 mM MgCl_2_, 10 mM MES, 150 µM acetosyringone), followed by syringe infiltration into 4- to 5-week-old *N. benthamiana* leaves at OD_600_ = 0.6. The inoculated *N. benthamiana* were incubated at 28 °C in the dark for 2 d and then at 28 °C under dark/light conditions (12 h/12 h) for 5 d. The stomatal aperture of the inoculation site was observed using an optical microscope after 3 h of light treatment. The vector 35S::GFP was used as the control. Each treatment was performed in three replicates.

## 3. Results

### 3.1. Differences in Structural Resistance between Sugarcane ZZ9 and GT42

**Figure 1:**
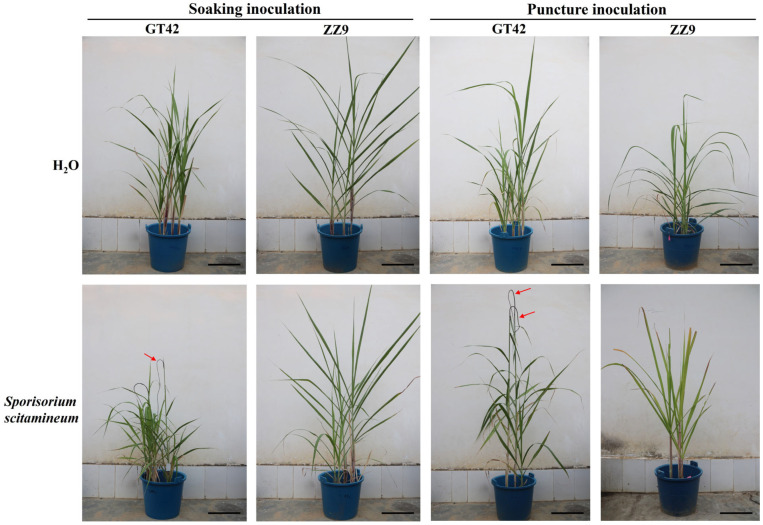
Smut resistance evaluations were conducted on the smut-susceptible sugarcane variety GT42 and the smut-resistant variety ZZ9, following 100 d of either soaking or puncture inoculation with *Sporisorium scitamineum*. Sugarcane inoculated with H_2_O was considered the control. Red arrows indicate black whips. Bars = 30 cm.

Five sugarcane varieties (smut-resistant: ZZ1, ZZ6, and ZZ9; smut-susceptible: GT42 and ROC22) were selected for this study. Previous research reports that GT42 and ROC22 exhibit higher smut incidence in the field, while ZZ1, ZZ6, and ZZ9 do not show symptoms of black whip [33]. To identify the resistant mechanisms (structural, biochemical, or physiological resistance), two smut inoculation methods (soaking and puncture) were employed on GT42 and ZZ9 [4]. Following three months of soaking inoculation with *S. scitamineum*, the susceptible sugarcane GT42 displayed smut symptoms, the while resistant sugarcane ZZ9 did not. In contrast, sugarcane GT42 demonstrated a higher incidence of smut when subjected to puncture inoculation, while ZZ9 also exhibited minimal black whip formation. Regardless of the inoculation method used, the smut incidence of ZZ9 was significantly lower than that of GT42 (Figure 1 and Table 1). These results indicate that structural resistance may play an important role in protecting sugarcane ZZ9 against smut.

### 3.2. Differences in the Stomatal Characteristics of Buds among Smut-Resistant and Smut-Susceptible Sugarcane Varieties

**Figure 2:**
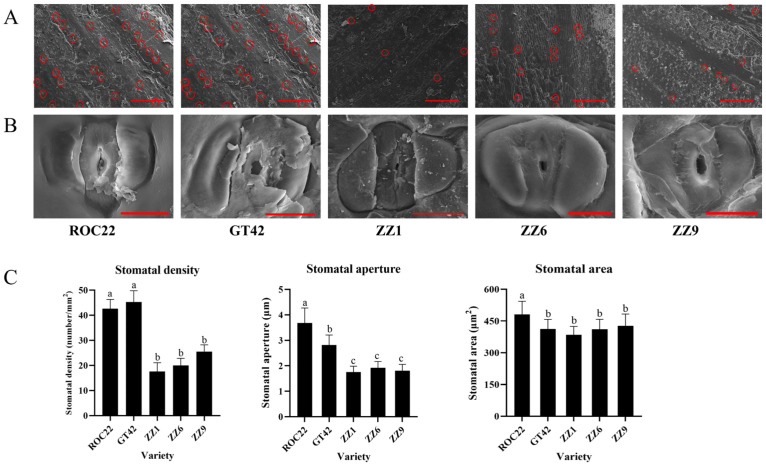
Observation and quantification of stomatal density, aperture, and area on sugarcane buds of different varieties. (**A**) Observation of stomatal density on sugarcane buds. Red circles indicate stomata on sugarcane buds. Bars = 200 μm. (**B**) Observation of stomata on sugarcane buds. Bars = 10 μm. (**C**) Statistical results of stomatal density, aperture, and area of outermost bud scales of sugarcane. Values followed by different letters are significantly different by Tukey’s test (*p* < 0.05). Three biological replications were performed.

To explore the role of the stomata in structural resistance to smut, SEM analysis was used to compare the differences in the stomatal characteristics of sugarcane buds among smut-resistant and smut-susceptible varieties. There were obvious differences in stomatal density between smut-susceptible (ROC22 and GT42) and smut-resistant (ZZ1, ZZ6, and ZZ9) varieties (Figure 2A), and there was no significant difference in stomatal morphology (Figure 2B). The quantification results showed that the stomatal density and aperture of smut-susceptible varieties were significantly higher than those of smut-resistant varieties. Additionally, ROC22 exhibited a larger stomatal area compared to ZZ1, ZZ6, ZZ9, and GT42 (Figure 2C). These results suggest that the stomatal characteristics of buds may be related to sugarcane smut resistance.

### 3.3. S. scitamineum Enters Sugarcane through the Stomata

**Figure 3:**
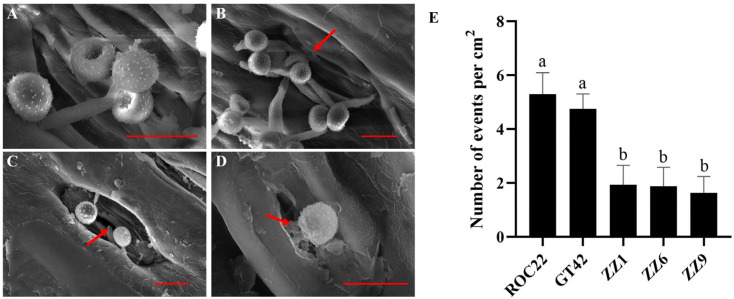
Infection of *S. scitamineum* through the stomata on the outermost bud scale of sugarcane. (**A**–**D**) Observations of *S. scitamineum* infection through the stomata on the outermost bud scale of sugarcane. Arrows indicate the germ tube of germinated smut teliospores. Bars = 10 μm. (**E**) Statistics of *S. scitamineum* infection events in the stomata per cm^2^. Values followed by different letters are significantly different by Tukey’s test (*p* < 0.05). Ten biological replications were performed.

To investigate the ability of *S. scitamineum* to invade sugarcane through the stomata of buds, the surface of buds of ROC22, GT42, ZZ1, ZZ6, and ZZ9 were observed with SEM after 1 d of *S. scitamineum* inoculation. As shown in Figure 3A, three *S. scitamineum* teliospores landed on the stomatal opening, one of which had germinated. However, it was not possible to determine whether the other two teliospores had germinated or not. In Figure 3B, several germinated teliospores are located adjacent to the stomata, with one of the germ tubes entering the stomata (Figure 3C,D). We quantified the occurrence of *S. scitamineum* invasion events through the sugarcane stomata and found that the number of events in smut-susceptible varieties (ROC22 and GT42) was significantly higher than that in smut-resistant varieties (ZZ1, ZZ6, and ZZ9) with the same area (Figure 3E). The results indicate that *S. scitamineum* could enter sugarcane through the bud stomata.

### 3.4. Differentially Expressed Stomata-Related Gene Analysis in GT42 and ZZ9

To identify important stomata-related genes that play a role in resistance to smut pathogens, RNA-seq of the outermost scales from the smut-susceptible sugarcane GT42 and smut-resistant sugarcane ZZ9 was conducted. A total of 39.92 GB of data was obtained, with read numbers ranging from 19,556,681 to 24,012,438 and Q30 values exceeding 92.63% for all samples (Table S2). This indicates high sequencing quality for the subsequent analysis. A total of 100,056 unigenes were obtained, with an average length of 856.13 bp (Table S3).

**Figure 4:**
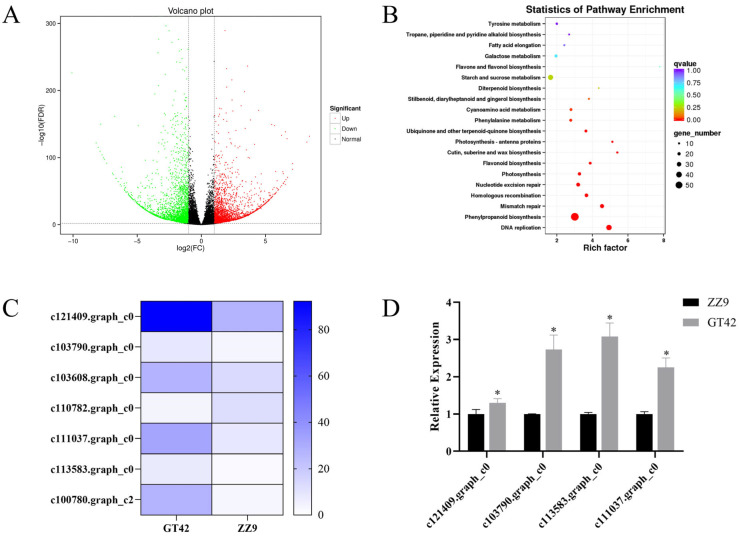
RNA-seq analysis of sugarcane GT42 and ZZ9 buds. (**A**) Volcano plot of differentially expressed genes (DEGs). There were 2769 upregulated and 3137 downregulated genes in GT42. (**B**) KEGG enrichment analysis of DEGs. (**C**) Expression profiles of *Expansin* genes in GT42 and ZZ9. (**D**) RT-qPCR analysis of four stomatal aperture-related gene expression levels in GT42 and ZZ9. GAPDH was used as internal standard. Three biological replications were conducted. * *p* < 0.05.

Compared with ZZ9, the expressions of 2769 genes in GT42 were upregulated, while those of 3137 genes were downregulated (Figure 4A). These genes were enriched in pathways of DNA replication, mismatch repair, etc. (Figure 4B and Table S4). Among them, DEGs of seven stomatal aperture-related genes encoding Expansin proteins [23], including c121409.graph_c0, c103790.graph_c0, c103608.graph_c0, c110782.graph_c0, c111037.graph_c0, c113583.graph_c0, and c100780.graph_c2, were identified. Six of these genes were upregulated in GT42 (Table 2 and Figure 4C), whose stomatal aperture was higher than that of ZZ9. Furthermore, the expressions of four genes (c121409.graph_c0, c103790.graph_c0, c113583.graph_c0, and c111037.graph_c0) were verified by RT-qPCR analysis. Consistent with the findings of RNA-seq, these four genes were all significantly upregulated in GT42 compared with ZZ9 (Figure 4D). The results imply that the expression of genes encoding Expansin proteins may be negatively related to smut resistance in sugarcane.

### 3.5. The Transient Overexpression of Two Expansin Protein-Encoding Genes Affected the Stomatal Aperture of N. benthamiana

**Figure 5:**
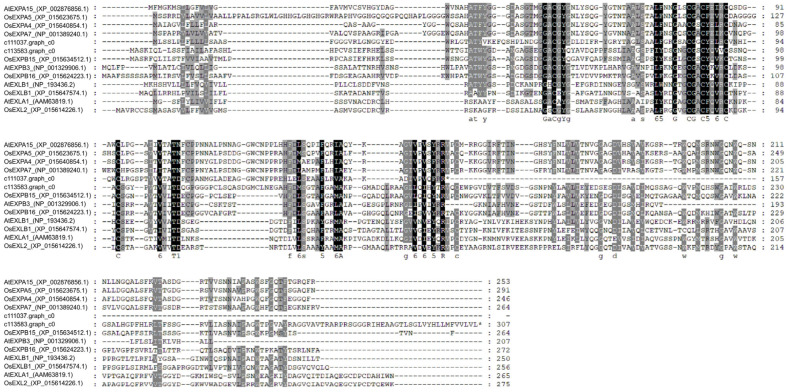
Sequence alignment analysis of c111037.graph_c0 and c113583.graph_c0. The Expansin protein sequences of *Oryza sativa* and *Arabidopsis thaliana* were used for analysis using MEGA 7. Black shades indicate that all proteins have the same amino acids. Grey shades indicate that some proteins have the same amino acids.

**Figure 6:**
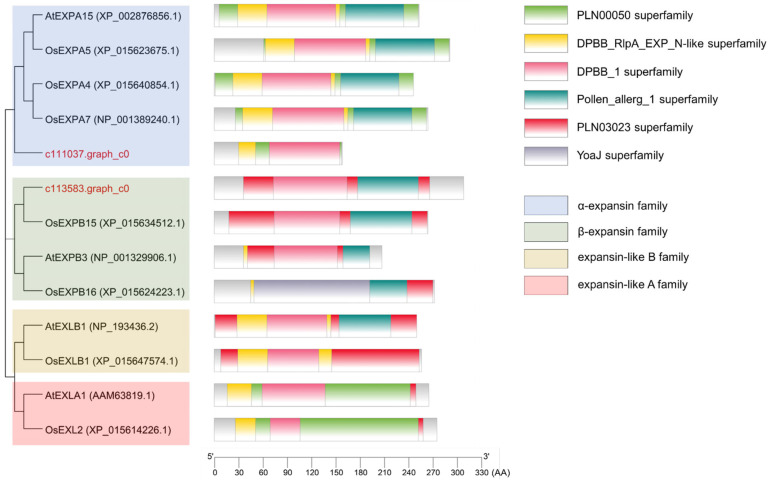
Phylogenetic tree analysis of c111037.graph_c0 and c113583.graph_c0. c111037.graph_c0 and c113583.graph_c0 belong to α-Expansin and β-Expansin, respectively. The domains were predicted by the Batch CD-search Tool.

**Figure 7:**
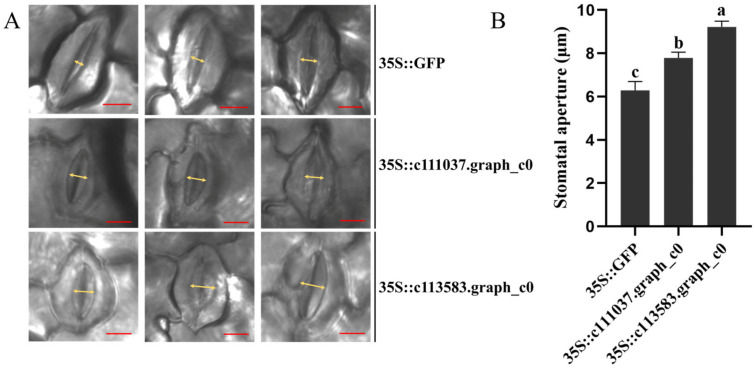
The transient expression of c111037.graph_c0 and c113583.graph_c0 increased the stomatal aperture on *Nicotiana benthamiana* leaves. (**A**) Observations of the stomatal aperture after the transient expression of c111037.graph_c0 and c113583.graph_c0. 35S::GFP was considered the control. Yellow arrows indicate the stomatal aperture. Bars = 10 μm. (**B**) Statistics of the stomatal aperture after the transient expression of c111037.graph_c0 and c113583.graph_c0. The maximum distance of the stomatal opening was quantified using ImageJ v1.8.0 and taken as the stomatal aperture. Values followed by different letters are significantly different by Tukey’s test (*p* < 0.05). Twelve biological replications were performed.

Based on the DEG analysis, we selected c111037.graph_c0 and c113583.graph_c0, with a complete protein domain, for further analysis to determine the role of *Expansin* protein-encoding genes in stomatal aperture. Figure 5 presents the conserved amino acids of c111037.graph_c0 and c113583.graph_c0 in Expansin proteins, belonging to the α-Expansin and β-Expansin families, respectively (Figure 6). To determine the role of *Expansin* protein-encoding genes in stomatal aperture and disease resistance, two *Expansin* protein-encoding genes (c111037.graph_c0 and c113583.graph_c0) (Figure S1) were transiently expressed in *N. benthamiana* leaves by agroinfiltration. After 35S::*GFP* (control), 35S::c111037.graph_c0, and 35S::c113583.graph_c0 transient expression in *N. benthamiana*, the stomatal aperture on *N. benthamiana* abaxial leaves was determined as 6.28 ± 1.41, 7.78 ± 0.92, and 9.21 ± 0.93 μm, respectively (Figure 7A). The stomatal aperture of *N. benthamiana* abaxial leaves treated with 35S::c111037.graph_c0 and 35S::c113583.graph_c0 was significantly higher than that of leaves treated with 35S::*GFP* (Figure 7B), indicating that c111037.graph_c0 and c113583.graph_c0 could affect the stomatal aperture.

## 4. Discussion

Smut resistance mechanisms include structural, biochemical, and physiological resistance [4,5]. At present, the structural resistance of smut remains largely unknown. *S. scitamineum*, the smut causal agent, enters sugarcane through buds. Therefore, we investigated the relationship between bud structure and smut resistance.

Previous research has associated sugarcane bud characteristics with sugarcane smut resistance, including bud size, bud trichomes, bud shape, etc. [4,9,34,35,36]. Furthermore, some secondary metabolites accumulated on buds also contributed to smut resistance, such as wax, glycoside, and flavonoids [4,6]. Our results reveal that the stomata exhibit differences in their bud characteristics (stomatal aperture and density) across smut-resistant (ZZ1, ZZ6, and ZZ9) and smut-susceptible (ROC22 and GT42) sugarcane varieties. This implies that the stomatal characteristics of buds play a role in structural resistance to sugarcane smut. In our previous study, the bud size of smut-susceptible (ROC22 and GT42) varieties was significantly higher than that of smut-resistant (ZZ1, ZZ6, and ZZ9) varieties [6]. This suggests that the total number of stomata per bud of GT42 and ROC22 is far more than that of ZZ1, ZZ6, and ZZ9, resulting in a greater risk of *S. scitamineum* infection from the stomata on buds. Moreover, the stomatal aperture of smut-susceptible (ROC22 and GT42) varieties was higher than that of smut-resistant (ZZ1, ZZ6, and ZZ9) varieties, also inducing a higher risk of *S. scitamineum* infection via the stomata.

Stomata are reported to play a role in pathogen invasions and plant defense, in cases such as *P. striiformis*-affected wheat [15] and downy mildew-affected grapevine [18]. In this study, although the germ tube of *S. scitamineum* was observed to invade sugarcane from the stomata, it did not occur frequently, with 1.6–5.3 events per cm^2^. This indicates that *S. scitamineum* infection via the stomata is a rare event and the dominant *S. scitamineum* infection pathway is appressorium formation [2,8]. However, infection from the stomata offers an easier entrance into sugarcane tissue compared to appressorium formation.

We conducted RNA-seq to identify the genes controlling the stomatal characteristics of buds. Plant stomatal development is mainly regulated by three types of *bHLH* transcription factors, namely, *SPCH*, *MUTE*, and *FAMA* [20,21,22]. Our RNA-seq results revealed two genes encoding SPCH and one gene encoding MUTE. These genes did not exhibit different expressions in ZZ9 and GT42, implying that there are other genes controlling the stomatal development of buds, and further study should be conducted. The stomatal aperture is controlled by various factors and genes [37]. Here, we selected the genes encoding *Expansin* proteins for further study, which can directly affect the stomatal aperture [23]. Seven genes encoding *Expansin* were identified from RNA-seq, six of which were upregulated in GT42. Two of these genes—with a complete protein domain (c111037.graph_c0 and c113583.graph_c0)—were employed to conduct further analysis. The results showed that they belong to the α-Expansin and β-Expansin families, which are known to have cell-wall loosening activity and participate in cell expansion [38], including guarding the cells of the stomata. The transient expression results revealed that they increased the opening of the stomata on *N. benthamiana* leaves. In summary, these findings indicate that these two genes encoding *Expansin* proteins induced a higher stomatal aperture in GT42 than in ZZ9. This may be because they loosened the guard cells of the stomata on the GT42 buds.

In conclusion, *S. scitamineum* could enter the sugarcane tissue via the stomata. Significant differences in the stomatal density and aperture of buds were observed between the smut-resistant and smut-susceptible varieties. Two genes encoding *Expansin* proteins could affect the stomatal aperture, possibly causing a higher stomatal aperture in GT42 than in ZZ9. Our results deepen the understanding of structural resistance mechanisms against sugarcane smut.

## Figures and Tables

**Table 1:** Smut incidence of GT42 and ZZ9 using soaking and puncture inoculation.

Variety	Method	No. of Plantlets Inoculated	No. of Whips	Smut Incidence (%)
GT42	Soaking inoculation	50	23	46
	Puncture inoculation	15	9	60
ZZ9	Soaking inoculation	50	0	0
	Puncture inoculation	15	1	6.67

**Table 2:** Statistics of differential expression of stomata-related genes.

Gene ID	GT42	ZZ9	log_2_FC	FDR	Description
c121409.graph_c0	92.516667	28.156667	1.0440377	9.27 × 10^−51^	*Expansin*-B11
c103790.graph_c0	8.4166667	3.2266667	1.4529291	1.1 × 10^−9^	*Expansin*-like A4
c103608.graph_c0	26.753333	12.603333	1.1398525	6.26 × 10^−14^	*Expansin*-B16
c110782.graph_c0	3.6666667	11.506667	−1.413006	2.65 × 10^−12^	*Expansin*-like A2
c111037.graph_c0	31.573333	8.6633333	1.7396968	7.39 × 10^−28^	*Expansin*-A16
c113583.graph_c0	7.3566667	2.0033333	1.6267807	3.89 × 10^−18^	*Expansin*-B12
c100780.graph_c2	27.056667	3.23	2.7556978	2.76 × 10^−29^	*Expansin*-B7

## Data Availability

The raw data generated during the current study are deposited in the Genome Sequence Archive at the National Genomics Data Center, China National Center for Bioinformation/Beijing Institute of Genomics, Chinese Academy of Sciences (GSA: CRA006705), and are publicly available online: https://ngdc.cncb.ac.cn/gsa, accessed on 7 June 2024.

## Supplementary Materials

The following supporting information can be downloaded at: https://www.mdpi.com/article/10.3390/jof10090631/s1, Figure S1: Construction of 35S::c111037.graph_c0 and 35S::c113583.graph_c0 vectors; Table S1: The primers used in this study; Table S2: Statistics of RNA sequencing; Table S3: Statistics of full-length distribution of sequencing fragments; Table S4: KEGG enrichment analysis of differentially expressed genes.
