# Supplementary material for: Two Sugarcane Expansin Protein-Coding Genes Contribute to Stomatal Aperture Associated with Structural Resistance to Sugarcane Smut
Source: J Fungi (Basel). 2024 Sep 3;10(9):631. doi: 10.3390/jof10090631 (PMC11433316; doi:10.3390/jof10090631)
Supplement: Supplementary file 1 [file jof-10-00631-s001.zip › Supplementary File S2.pdf]

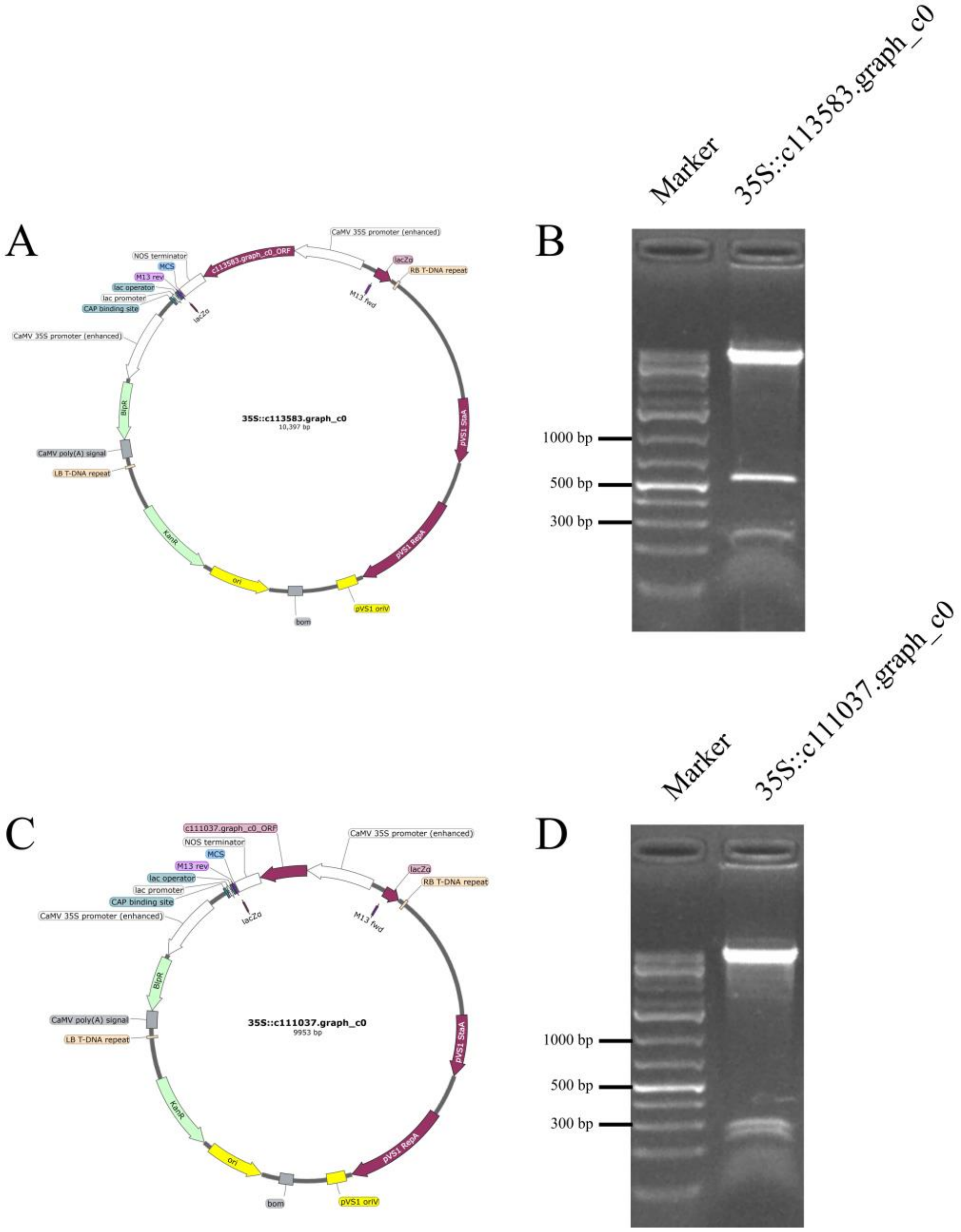

Figure S1. Construction of 35S::c111037.graph_c0 and 35S::c113583.graph_c0 vectors. The maps of 35S::c111583.graph_c0 and 35S::c113037.graph_c0 were shown in (**A**) and (**C**), respectively. The enzyme digestion validation (BamH Ⅰ and Sac Ⅰ) of 35S::c111583.graph_c0 and 35S::c113037.graph_c0 were shown in (**B**) and (**D**), respectively.
